# Pulmonary Veno-Occlusive Disease: A Rare Cause of Pulmonary Hypertension

**DOI:** 10.1177/2324709619840375

**Published:** 2019-04-22

**Authors:** Said Hajouli, Muhamad Alhaj Moustafa, Jessica S. Wang Memoli

**Affiliations:** 1 MedStar Washington Hospital Center, Washington, DC, USA

**Keywords:** pulmonary veno-occlusive disease, pulmonary hypertension, hypoxia, lung transplantation, ventilation-perfusion lung scan

## Abstract

Pulmonary veno-occlusive disease (PVOD) is a rare entity that is usually mistaken with pulmonary arterial hypertension (PAH) but is considered class I′ of PAH. It is important to subclassify PVOD and distinguish it from PAH as treatment with vasodilators in PVOD patients is controversial and may be fatal. In this article, we describe a case of PVOD and how we diagnosed it.

## Introduction

Pulmonary veno-occlusive disease (PVOD) is a rare and fatal cause of pulmonary hypertension (PH) that is difficult to diagnose and treat. First described in 1934 by Dr Julius Hora of the University of Munich,^1^ PVOD is characterized by widespread occlusion of the pulmonary venules by fibrous tissue. The reported incidence of PVOD is 0.1 to 0.2 cases per million every year.^2^ Patients usually present with nonspecific respiratory and/or cardiac symptoms, including shortness of breath, dyspnea on exertion, fatigue, chest pain, dizziness, cough, and hemoptysis. PVOD is a clinicopathological entity with the diagnosis based on clinical manifestations and tissue confirmation. A combined picture of pulmonary artery hypertension (PAH) along with concurrent pulmonary edema findings on diagnostic imaging is usually the initial hint toward PVOD diagnosis. The gold standard to confirm the diagnosis is lung biopsy, which usually shows pulmonary venules intimal fibrosis with diffuse smooth muscle narrowing, post-capillary proliferation, and interstitial/alveolar hemosiderophages.^3^ Biopsy is not commonly performed because of the bleeding risk in the setting of fragile pulmonary veins.^4^ PVOD has a very poor prognosis and lung transplantation is the best available treatment.^5^ In this article, we present a case of PVOD masquerading as chronic thromboembolic PH (CTEPH) and how the patient was diagnosed and managed.

## Case Presentation

**Figure 1.**
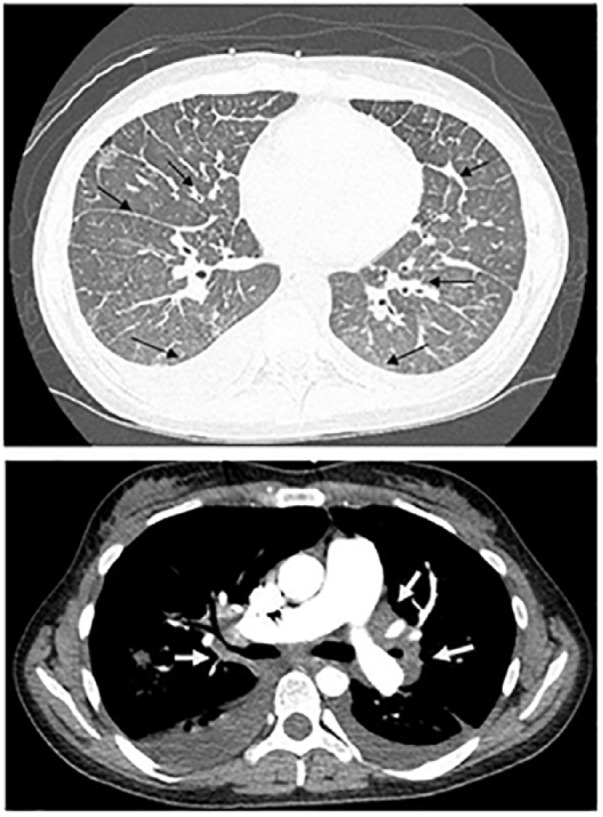
CT scan of the chest showing bilateral hilar and mediastinal lymph-adenopathy (white arrows), focal areas of increased interstitial thickening scattered throughout both lungs, bilateral pleural effusions, prominence of the interlobular septa (black arrows), and the absence of pulmonary embolism.

**Figure 2.**
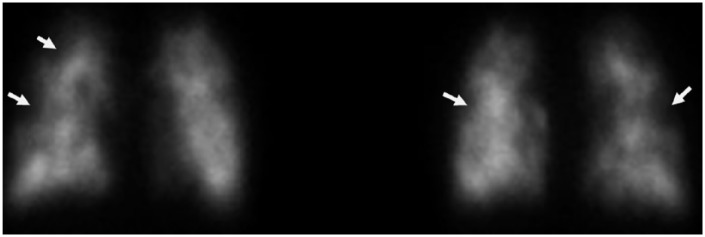
V/Q scan showing high probability for PE. Innumerable small sized peripheral and non-peripheral perfusion defects scattered throughout the entirety of bilateral lungs. Several perfusion/ventilation mismatch areas are seen.

A 33-year-old Caucasian woman with history of unconfirmed pulmonary sarcoidosis presented to our emergency department with a 1-month duration of progressive shortness of breath. In the emergency department, she was tachypneic and hypoxic to 88% oxygen saturation on 8 L of supplemental oxygen. Chest X-ray was consistent with pulmonary venous congestion. Bi-level positive airway pressure and diureses with intravenous furosemide was started. Computed tomography (CT) pulmonary angiogram was negative for pulmonary embolism (PE) but showed ground glass opacities, hilar and mediastinal lymphadenopathy, bilateral pleural effusions, and increased prominence of the interlobular septa (Figure 1). Echocardiogram showed evidence of severe pulmonary hypertension with estimated pulmonary artery pressure of 85 to 90 mm Hg, normal left ventricle, dilated right ventricle and right atria, and severely decreased right ventricle systolic function. Right heart catheterization showed normal filling pressures and pulmonary capillary wedge pressure but elevated pulmonary artery pressure and pulmonary vascular resistance ( Table 1). Laboratory workup was negative for HIV, antinuclear antibody, abnormal thyroid stimulating hormone, rheumatic factor, ANCA, anti-SCL70, or elevated erythrocyte sedimentation rate (Table 2). Pulmonary function tests (PFTs) showed normal lung volumes with severely decreased diffusing lung capacity for carbon monoxide (DLCO). Ventilation/perfusion lung scan (V/Q scan) showed perfusion defects scattered throughout the entirety of bilateral lungs with several areas of perfusion/ventilation mismatch (Figure 2), which raised the suspicion of CTEPH, and patient was started on heparin infusion. Lower extremities duplex was negative for acute or chronic deep venous thrombosis and a repeat CT pulmonary angiogram showed findings as mentioned above and no PE. Those CT findings were not consistent with CTEPH. CTEPH CT usually shows disparity in segmental arteries size, calcifications/dilatation of central pulmonary arteries, mosaic perfusion, and enlarged bronchial arteries. Bronchial dilatation without bronchial wall thickening is the most specific CT feature for CTEPH.^6,7^ Small peripheral PEs should not cause that severe PH without these CTEPH CT findings. CTEPH was ruled out and heparin infusion was stopped. With the previous clinical diagnoses of pulmonary sarcoidosis and the current chest CT findings, sarcoidosis remained in the differential for the etiology of her PH. Bronchoalveolar lavage showed hemosiderin-laden macrophages, but no diffuse alveolar hemorrhage. Transbronchial biopsy had focal mild fibrosis with no carcinoma or granuloma, and endobronchial ultrasound guided transbronchial needle aspiration also did not show carcinoma or granuloma. All microbiology was negative. Based on the above-mentioned results, sarcoidosis was less likely. The patient had mild improvement with diuresis and remained hypoxic after 8 days of hospitalization requiring 3 liters of oxygen on nasal cannula.

**Table 1.** Right Heart Catheterization Results.

Right atria pressure	2 mm Hg
Right ventricular pressure	80/5 mm Hg
Pulmonary artery pressure	80/35 mm Hg
Mean pulmonary artery pressure	52 mm Hg
Pulmonary capillary wedge pressure	8 mm Hg
Pulmonary vascular resistance	951 D/S
Fick cardiac output	3.2 L/min
Fick Cardiac Index	2.1 L/min/m^2^

**Table 2.** Laboratory Results.

White blood cells	10.3 K/UL
Hemoglobin	11.6 g/dL
Hematocrit	36.1%
Platelet	313 K/UL
Sodium	138 µmol/L
Potassium	3.5 µmol/L
Chloride	105 µmol/L
CO_2_	23 µmol/L
BUN	15 MH/dL
Creatinine	0.82 mg/dL
Glucose	133 mg/dL
Anti-nuclear antibody	1:40
Rheumatic factor	<1:16
Thyroid stimulating hormone	2.5 mLU/L
HIV	Negative
Erythrocyte sedimentation rate	10 mm/h
Antineutrophil cytoplasmic autoantibody	<1:20
Anti-SCL 70	20 units/mL

Abbreviations: BUN, blood urea nitrogen; HIV, human immunodeficiency virus.

Based on the above-mentioned findings, a clinical diagnosis of PVOD was suspected. The patient was referred to another facility for lung transplantation evaluation. The patient was to be admitted to the coronary care unit for close monitoring and titration of epoprostenol while being evaluated for lung transplant. Unfortunately, patient died before initiation of medical therapy or lung transplantation evaluation.

## Discussion

PVOD is a rare subtype of PAH group I called group I′.^8,9^ In PVOD, there is a post-capillary PH due to venules occlusion while pre-capillary pulmonary hypertension is the cause of PAH. It is estimated that 5% of patients diagnosed with group I PAH have PVOD as clinical and hemodynamic features are similar.^10^ In one study, 12 of 14 patients with a diagnosis of PAH who failed medical therapy had PVOD on lung tissue examinations.^11^ It is critical to distinguish PVOD from PAH as treating PVOD with vasodilators may lead to fatal pulmonary edema and death.^12^ The pathologic hallmark of PVOD is diffuse occlusion of the post-capillary pulmonary venules by fibrous tissue leading to intimal thickening and smooth muscle hypertrophy.^3,13^ The cause of PVOD is unknown but could result from a multiplicity of possible insults including viral infections like HIV,^14^ genetic causes as in EIF2AK4 gene mutations,^15^ thrombotic diathesis,^16^ hematologic malignancies, peripheral blood stem cell transplantation,^17^ bone marrow transplantation,^18^ toxic exposure as in chemotherapies,^19^ and autoimmune disorders including sarcoidosis, Hashimoto’s thyroiditis, systemic lupus erythematosus, rheumatoid arthritis, mixed connective tissue disease, and celiac disease.^20-22^

The diagnostic workup includes right heart catheterization to confirm pulmonary hypertension and increased pulmonary vascular resistance with normal pulmonary capillary wedge pressure and filling pressures. PFTs usually show normal spirometry and lung volumes with severe hypoxemia and severely decreased DLCO. Interstitial edema in PVOD patients leads to poor membrane diffusion and low DLCO. DLCO of <55% has a specificity of 89.5% and a sensitivity of 64.3% for the diagnosis of PVOD in a case series of 24 cases of histology-confirmed PVOD patients (comorbid patients like sarcoidosis and HIV were excluded from the study and they included only severe cases of PVOD as they required histologic confirmation of PVOD, which was done after lung transplantation or autopsy)^23^ Severely decreased DLCO can also be found in connective tissue-related PH-like systemic sclerosis^24^ and some cases of idiopathic pulmonary arterial hypertension,^25^ which should be ruled out first. High-resolution CT scan of the chest usually shows bilateral pleural effusions, ground glass opacities, hilar and mediastinal lymphadenopathy, and interlobular septal thickening.^26^ These CT scan findings are classic for the PVOD after more common conditions like sarcoidosis, pneumoconiosis, and parenchymal lung diseases have been ruled out. They are typically not seen in patients with idiopathic PAH or CTEPH.^27^ These CT scan findings have 89% specificity, 95.5% sensitivity, 98.5% negative predictive value, and 72.5% positive predictive value, with a diagnostic accuracy of 90.5% in PVOD patients.^28^ In most cases, V/Q scan will be normal with 73% of cases having normal perfusion and 86% having normal ventilation.^29^ While V/Q mismatch in PVOD patients is rare, it may lead to the misdiagnosis of PVOD as CTEPH. European Respiratory Society/European Society of Cardiology guidelines suggested that nonmatched perfusion defects on V/Q scan in patients with PAH may suggest PVOD, but this suggestion was based on a series of only 3 PVOD cases (2 of them had a histological diagnosis of PVOD and the other one had a clinical diagnosis of PVOD),^30^ but recent studies showed that most PVOD patients will have normal V/Q scan according to a cohort study in 2012 that had 56 PVOD patients (25 cases had a clinical diagnosis of PVOD, 12 cases had a histological proof of PVOD, and 19 cases had a high probable PVOD diagnosis when developing pulmonary edema after initiation of a specific PAH therapy). Only 7.1% had V/Q mismatch (3 confirmed disease and 1 highly probable), and in most of the cases, the deficit was in the perfusion part of the V/Q scan.^29^ Our patient had severe PVOD that caused the V/Q mismatch. Chronic post-capillary pulmonary obstruction/hypertension in PVOD increases the hydrostatic pressures in the pulmonary capillaries and visceral pleural capillaries and can lead to transudation of fluids into the interstitium and pleural space causing Kerley B lines and pleural effusions seen in chest X-ray, which may lead to misdiagnose it as congestive heart failure. The presence of post-capillary blockage can lead to occult alveolar hemorrhage (characterized by hemosiderin macrophages in BAL),^31^ so it is not recommended to perform transbronchial lung biopsy to make the diagnosis given the high risk of life-threatening pulmonary bleeding secondary to frail pulmonary veins in PVOD. Ultimately, the diagnosis of PVOD can be made by the constellations of high clinical suspicion, severe hypoxia on ABG, severely decreased DLCO on PFTs, normal V/Q scan, severe pulmonary hypertension on right heart catheterization, and high-resolution chest CT findings of ground glass opacities, hilar and mediastinal lymphadenopathy, and interlobular septal thickening.^32^

There are no treatment guidelines for PVOD. Treatment other than lung transplantation consists of supportive measures with supplemental oxygen for hypoxia, diuretics for volume overload and right heart failure, and smoking cessation. Patients should receive routine vaccinations to prevent pneumococcal pneumonia and influenza. There are no outcome data on anticoagulation use in patients with PVOD. Anticoagulation can be used but with caution as some patients may have occult pulmonary hemorrhage.^33^ Advanced therapies for PAH in PVOD will dilate the pulmonary arterioles, and with the resistant fixed pulmonary veins, the trans-capillary hydrostatic pressure will increase and may cause massive pulmonary edema and respiratory failure.^5,23^ Thus, they should be used with caution and only as an urgent life-saving bridge to lung transplantation.

The prognosis of the PVOD is poor with estimated 1-year mortality of 72% without lung transplantation.^5^ The mean time from diagnosis to death or lung transplantation is 11.8 months in PVOD patients, and the mean time from first symptoms to death or lung transplantation is 24.4 months.^34^ Our patient had a more rapid progressive course with time from first symptom of shortness of breath to death being 6 months and the time from diagnosis to death was only 1 month.

The PVOD diagnosis in our patient was challenging because of the unusual scattered mismatch on the V/Q scan, which made us consider CTEPH. She also had previous clinical diagnosis of sarcoidosis based on CT chest findings. This necessitated the bronchoscopy. However, sarcoidosis was less likely to be the cause of her PH with no evidence of granuloma found. One limitation to this case is that no lung histology or autopsy to confirm the clinical diagnosis was obtained.

In summary, PVOD should be considered in all patients presenting with PAH, as early diagnosis and referral for lung transplantation can be lifesaving.
